# The Conversion of Post-traumatic Hip Fusion to Total Hip Arthroplasty: A Case Report

**DOI:** 10.7759/cureus.87407

**Published:** 2025-07-07

**Authors:** Noora Abuzeyad, Ahmed Al Khalifa, Faisal Falamarzi, Fahad Al Khalifa

**Affiliations:** 1 Orthopaedics, Royal Medical Services, Kingdom of Bahrain, Riffa, BHR; 2 Orthopaedic Trauma/Sports and Arthoplasty Surgery, Royal Medical Services, Kingdom of Bahrain, Riffa, BHR

**Keywords:** case report, conversion hip arthroplasty, hip fusion, post-traumatic, surgical intervention, total hip replacement

## Abstract

The goal of converting a hip fusion to a total hip arthroplasty (THA) is to improve mobility and function by replacing the fused joint with a more flexible and functional prosthetic implant. This complex procedure addresses challenges related to altered biomechanics and potential bone loss from the previous fusion. In this case report, we discuss the conversion of a post-traumatic hip fusion to THA in a 25-year-old female with debilitating pain and function impairment to movement. Clinical and plain radiographic findings are discussed. This report highlights the necessity of comprehensive preoperative assessments and individualized surgical strategies to optimize surgical outcomes in patients with prior hip fusions to improve their mobility, pain relief, and overall satisfaction.

## Introduction

Post-traumatic hip fusion is a procedure performed to manage severe hip injuries in young, healthy individuals, providing stability and pain relief [1,2]. However, over time, patients might experience persistent back, contralateral hip, or knee pain, along with functional impairments [3], necessitating a conversion to total hip arthroplasty (THA). The complexities associated with converting a fused hip to THA, such as the loss of surgical landmarks and the presence of scar tissue [4,5], can complicate the surgical procedure. Nevertheless, it can significantly improve their symptoms and restore their mobility [6]. This case report describes the successful conversion of a post-traumatic hip fusion to THA in a Middle Eastern female. We present the patient's clinical and radiological findings and highlight key surgical considerations pre-, intra-, and postoperatively, thereby contributing valuable insights to the existing literature.

## Case presentation

A 25-year-old female presented with persistent left hip pain and limited mobility following a traumatic left hip fusion in 2014 due to an acetabulum fracture and posterior dislocation of the left femur from a road traffic accident. The hip fusion had been performed at another facility, and although the exact technique was unclear, the preoperative X-ray and scar suggested that internal fixation had been used. In 2017, she had undergone another surgical intervention to excise heterotopic calcification, which had led to sciatic nerve palsy. In 2020, she had experienced a periprosthetic fracture in the left femur shaft after a slip-and-fall accident and undergone open reduction and internal fixation (ORIF) of the proximal femur using a dynamic compression plate (DCS). The DCS plate had been selected due to its ability to provide stable compression for diaphyseal fractures and avoid interference with previous hardware. After years of constant pain and limited range of motion hindering daily activities, she had opted for conversion to THA in 2024 to address her ongoing hip issues and improve her quality of life.

**Figure 1:**
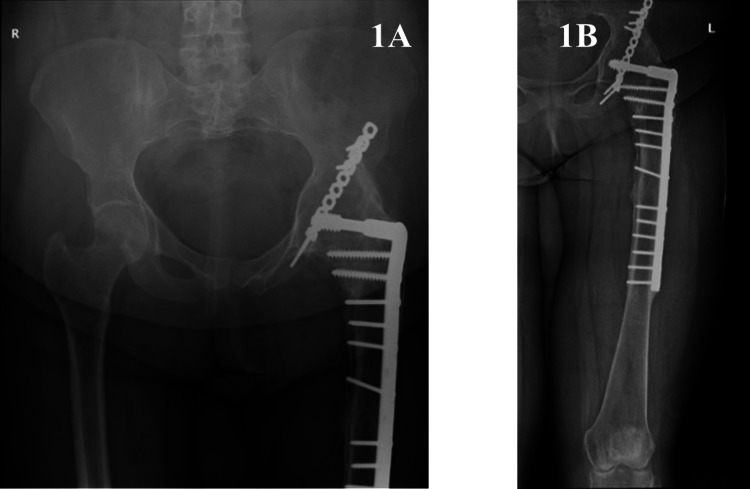
Preoperative plain radiographs of the left hip joint Preoperative plain radiograph of the left knee joint in anterior views showing hip arthrodesis and open reduction and internal fixation of the left proximal femur

On physical examination, the patient displayed generalized muscle atrophy in the left lower limb. There were no signs of inflammation, swelling, or changes in the overlying skin. Despite the previous hip fusion, hip motion was assessed by observing any residual or compensatory movements from adjacent joints, such as the lumbar spine or pelvis, during clinical examination. Hip flexion was limited to 25°, with up to 5° hip extension. Hip abduction was restricted to 25°, and hip adduction was 40°. Hip rotation was severely limited. The patella was mobile, and there was flicker movement in the ankle joint with muscle strength rated at 2/5. The patient demonstrated a foot drop, with power assessed at 0/5, and plantarflexion strength at 4/5. Vascular assessment revealed intact pulses with a capillary refill of less than two seconds. Sensation was absent over the dorsum and lateral aspect of the foot, but intact on the medial aspect. A mild leg length discrepancy of about 2.5 cm was noted. Plain radiographs in anteroposterior and lateral views revealed the presence of left hip arthrodesis (Figures 1A, 1B).

**Figure 2:**
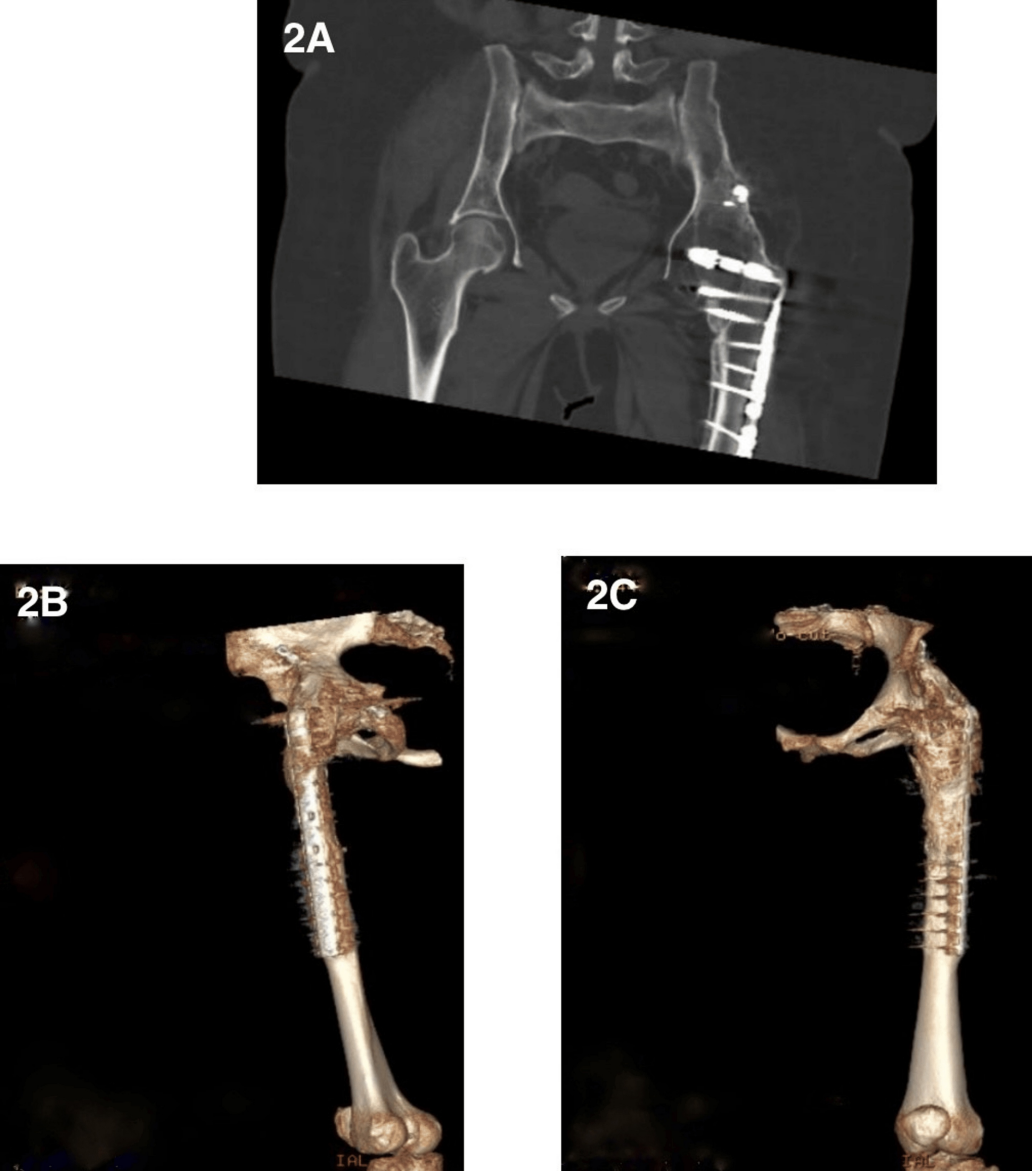
CT of the left hip joint Preoperative CT scan showing the core pelvis (A) and a 3D view of the left femur (B/C), confirming findings of the plain radiographs - hip arthrodesis and open reduction and internal fixation of the left proximal femur CT: computed tomography

After a thorough discussion about treatment options, the patient opted for hardware removal and conversion to left THA. Preoperative CT was performed (Figures 2A, 2B, 2C) due to the patient's history of multiple prior surgeries, including a hip fusion performed at an outside institution. Detailed operative records were unavailable, and given the complexity of the case and altered anatomy, CT imaging was essential for accurate preoperative planning, better visualization of the fusion status, bone quality, and surrounding structures. Surgery was performed at the left lateral decubitus position under general anesthesia and the modified Hardinge approach was used to access the hip joint and remove the previous fixation (a total of 12 screws). The modified Hardinge approach was chosen due to its ability to provide direct and stable access to the hip joint, particularly in the context of previous acetabular ORIF and heterotopic ossification (HO).

During surgery, atrophy of the vastus lateralis, gluteus medius, and minimus was noted alongside the presence of heterotopic ossifications, which were excised. The previous hardware was completely removed, and a biopsy was sent for culture and sensitivity (C&S). We fixed the proximal femur provisionally with a locking plate to avoid iatrogenic femur fracture. Under fluoroscopic guidance, a careful osteotomy was done, and neck cuts were marked, starting with the most distal cut, just above the greater trochanter, and a wedge cut proximal to it, making sure to avoid the acetabulum. The acetabulum was prepared with serial reaming to a diameter of 47 mm, followed by the insertion of a stable 48 mm acetabular shell secured with a 20 mm posterior superior screw.

**Figure 3:**
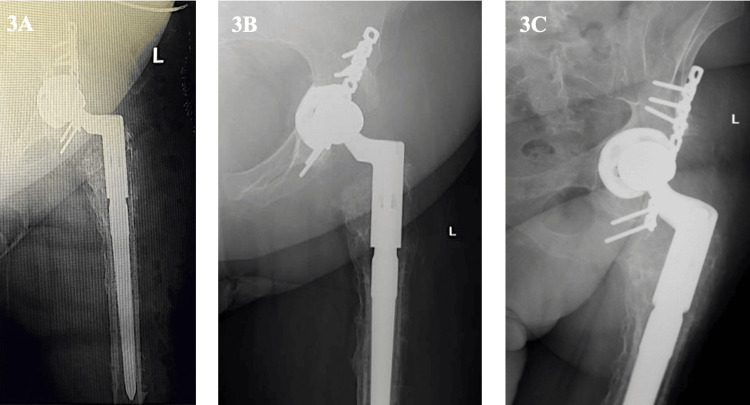
Postoperative plain radiographs of the left hip joint Postoperative plain radiographs of the left hip joint post conversion to total hip arthroplasty

Serial reaming of the femoral canal was conducted up to 20 mm. The final modular components used were a distal stem measuring 18 x 190 mm and a proximal stem measuring 20 x 85 mm, with a Wagner stem selected for femoral fixation. During the procedure, the acetabular cup was positioned with an anteversion angle of approximately 20°, based on intraoperative guidance. A 32 mm +1 head was successfully inserted as it provided a satisfactory balance between range of motion and stability, and the entire construct was reduced. The hip was stable in flexion, extension, and external rotation, and limb length was restored to equal. Postoperative X-rays are shown in Figure 3.

A week following surgery, the patient began physiotherapy with left hip flexion at 30° and abduction not assessed due to stiffness. Muscle testing showed strength levels of around 2/5 for the left hip and 3/5 for the left knee. Two months post-surgery, improvements were notable, with hip flexion reaching 70° and abduction improving to 25°. Muscle strength increased to 4/5 for the left hip and 3/5 for the left knee. The improvement in hip strength was most likely related to recovery in the iliopsoas (for flexion) and gluteus medius (for abduction). The knee strength improvement was likely due to the recovery of the quadriceps and hamstrings, which had been weakened due to disuse and previous surgeries. The patient has been advised to continue with physiotherapy according to her treatment plan.

## Discussion

Converting a hip fusion secondary to post-traumatic arthritis into a THA poses unique challenges, requiring meticulous preoperative planning, individualized surgical strategies, and comprehensive postoperative rehabilitation. This report highlights the importance of addressing the complexities associated with post-fusion patients, including a history of multiple surgeries, muscle atrophy, and sciatic nerve palsy. Existing literature supports the conversion of a hip fusion to a THA as an effective intervention for alleviating pain and improving function [7], even though it carries a higher risk of complications compared to primary THA [8]. In our case, a careful preoperative assessment of abductor muscle strength and radiological evaluation were crucial for achieving a favorable outcome. Howard et al. reported that conversion to THA leads to marked improvements in pain and leg length discrepancy, underscoring the transformative potential of this surgery [9]. Additionally, Parilla et al. observed that younger patients, particularly those under the age of 30, demonstrate favorable outcomes with clinical improvements sustained at 10 years postoperatively and implant survivorship extending to 15 years [10].

Our surgical approach utilized a modified Hardinge technique, which facilitated effective exposure and removal of the existing femoral hardware. This technique addressed the anatomical and technical complexities in post-fusion surgeries. The presence of heterotopic ossification [11] and muscle atrophy [12] in the patient represents common encounters in a conversion THR [13,14]. Additionally, the presence of a dysfunctional lower limb, compounded by previous surgeries and altered anatomy, further increases the likelihood of complications.

Conversion to THA is inherently associated with higher surgical risks compared to a primary THA, including increased likelihood of bleeding, infection, and neurovascular injuries. The risk of implant instability is heightened in these cases due to the lack of functional muscle stabilizers caused by the prior hip fusion and subsequent atrophy [15]. Furthermore, patients undergoing conversion THA typically experience a more prolonged recovery period compared to those undergoing primary THA, underscoring the need for comprehensive postoperative care and rehabilitation [16]. Moreover, the dysfunctional lower limb, in this case due to years of abnormal mechanics, muscle atrophy, and compensatory movement patterns following fusion and other surgeries, further complicates the recovery process. The altered anatomy and muscle weakness affect the patient’s ability to load the joint appropriately, making it harder to maintain long-term stability and proper gait.

Despite these challenges, the long-term outcomes of conversion to THA are encouraging. Joshi et al. reported implant survival rates of 96.1% at 10 years, 89.9% at 15 years, and 72.8% at 26 years, underscoring the durability and viability of this surgical approach [17]. Furthermore, Morsi found that clinical improvements were sustained for up to three years post-surgery, with most patients experiencing notable enhancements in mobility, including reduced reliance on walking aids [16]. While the recovery trajectory after conversion hip arthroplasty may demand an extended timeframe for evident enhancement, the procedure has demonstrated notable improvements in the range of motion and functional capacity in patients over time [18,19]. Effective patient education is integral to the success of conversion THA. Preoperative counseling regarding potential risks, expected outcomes, and the critical role of postoperative physiotherapy encourages realistic expectations and enhances patient satisfaction [20].

## Conclusions

For patients facing complications from a previous hip fusion, conversion to THA should be considered as a treatment option. The decision should be tailored to the individual patient’s baseline health status and functional goals. Advanced imaging techniques and surgical planning tools during the preoperative and intraoperative phases can significantly enhance the likelihood of a successful outcome. Additionally, comprehensive postoperative rehabilitation, including physical therapy, is essential for optimizing recovery and maximizing the long-term benefits of the procedure.
